# Comparative analysis of dosimetric parameters of three different radiation techniques for patients with Graves’ ophthalmopathy treated with retro-orbital irradiation

**DOI:** 10.1186/1748-717X-7-199

**Published:** 2012-11-26

**Authors:** Victor HF Lee, Sherry CY Ng, Cheuk Wai Choi, Mai Yee Luk, To Wai Leung, Gordon KH Au, Dora LW Kwong

**Affiliations:** 1 Department of Clinical Oncology, Li Ka Shing Faculty of Medicine, The University of Hong Kong, 1/F, Professorial Block, Queen Mary Hospital, 102 Pokfulam Road, Pokfulam, Hong Kong; 2 Department of Community Medicine, School of Public Health, Li Ka Shing Faculty of Medicine, The University of Hong Kong, 7 Sassoon Road, Hong Kong, Hong Kong

**Keywords:** Intensity-modulated radiation therapy, Graves’ ophthalmopathy, Retro-orbital irradiation

## Abstract

**Background:**

We would like to investigate the if IMRT produced better target coverage and dose sparing to adjacent normal structures as compared with 3-dimensional conformal radiotherapy (3DCRT) and lateral opposing fields (LOF) for patients with Graves’ ophthalmopathy treated with retro-orbital irradiation.

**Methods:**

Ten consecutive patients diagnosed with Graves’ ophthalmopathy were prospectively recruited into this study. An individual IMRT, 3DCRT and LOF plan was created for each patient. Conformity index (CI), homogeneity index (HI) and other dosimetric parameters of the targets and organs-at-risk (OAR) generated by IMRT were compared with the other two techniques.

**Results:**

Mann–Whitney U test demonstrated that CI generated by IMRT was superior to that produced by 3DCRT and LOF (p=0.005 for both respectively). Similarly HI with IMRT was proven better than 3DCRT (p=0.007) and LOF (p=0.005). IMRT gave rise to better dose sparing to some OARs including globes, lenses and optic nerves as compared with 3DCRT but not with LOF.

**Conclusions:**

IMRT, as compared with 3DCRT and LOF, was found to have a better target coverage, conformity and homogeneity and dose sparing to some surrounding structures, despite a slight increase but clinically negligible dose to other structures. Dosimetrically it might be a preferred treatment technique and a longer follow up is warranted to establish its role in routine clinical use.

## Introduction

Graves’ ophthalmopathy is an orbital inflammatory pathology associated with an underlying autoimmune thyroid disease particularly Graves’ disease [1,2]. Radiotherapy has long been used for this pathology, besides decompressive and corrective surgery as well as systemic steroids [1,3-5]. Traditionally a pair of lateral opposing fields (LOF) directed to the orbital structures has been adopted for decades in virtue of its easy set-up and prompt delivery. The beams are either blocked anteriorly or tilted 5 degrees posteriorly to minimize dose to the lenses. However, this will inevitably lead to inadequate dose to parts of the orbital structures especially the insertions of the extra-ocular muscles and the anterior portions of the retro-orbital fat. More advanced technique including 3-dimensional conformal radiotherapy (3DCRT), intensity modulated radiation therapy (IMRT) and robotic stereotactic radiotherapy are increasingly gaining popularity, due to their superior target coverage, dose escalation to the targets and better radiation sparing of normal structures [6-12]. In this study, we would like to investigate if IMRT provides a better target coverage as well as superior dose sparing to the normal structures as compared with 3DCRT and LOF for patients treated with orbital irradiation for their Graves’ ophthalmopathy.

## Methods

**Figure 1:**
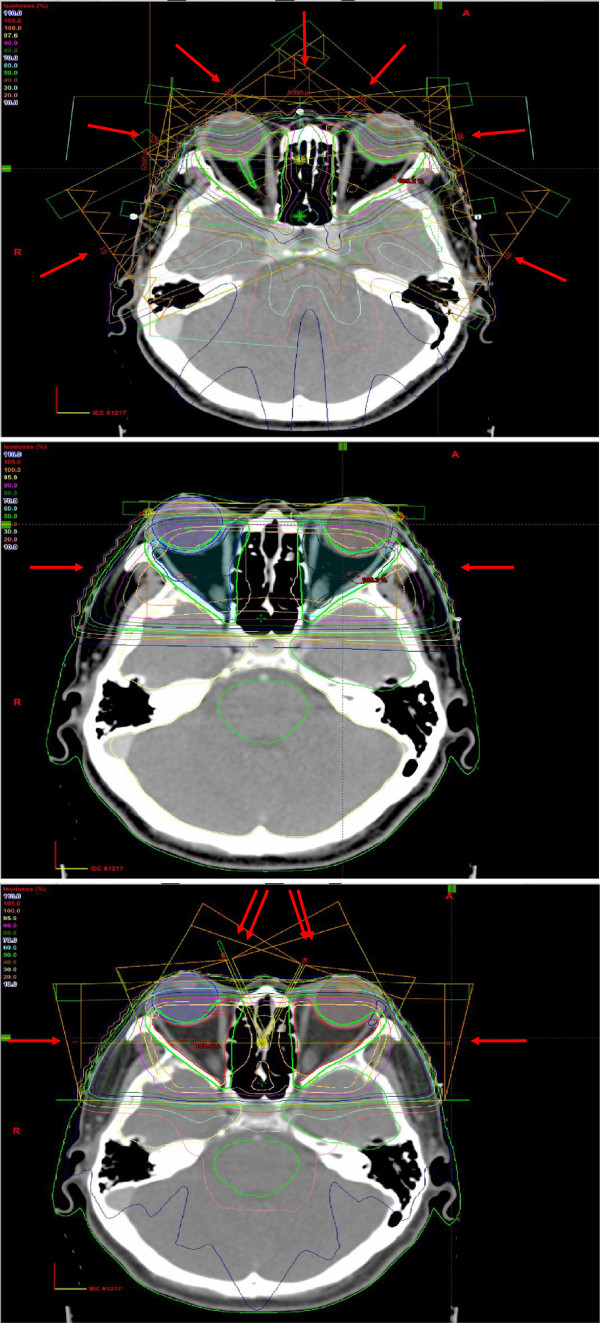
**Field arrangement by IMRT (uppermost), LOF (middle) and 3DCRT (lowermost).** Arrows indicate the directions of beams.

Ten consecutive patients with Graves’ ophthalmopathy diagnosed by ophthalmologists between July 2009 and June 2011 were treated with retro-orbital irradiation. After immobilized by custom-made thermoplastic cast, they underwent computed tomography (CT) scan with slice thickness 2.5mm for image acquisition and target contouring. The gross tumour volume (GTV) which was also equivalent to clinical target volume (CTV) included the retro-orbital fatty spaces together with the main bulk, origins and insertions of the extra-ocular muscles of both eyes. The globes, lenses, optic nerves, optic chiasm and lacrimal glands were outlined as organs-at-risk (OAR). A 2mm concentric margin around the GTV was created by boolean operators of the Eclipse Treatment Planning System version 8.9 (Varian Medical Systems, Palo Alto, CA) to generate the planning target volumes (PTV). All patients were given 10Gy in 10 fractions over 2 weeks delivered by reversely planned 7-field IMRT (directed at 0°, 40°, 80°, 110°, 250°, 280° and 320°), using anisotropic analytical algorithm with a 2.5mm calculation grid as planned by our treatment planning system with objective functions to minimize the square of dose difference between the constraints of the actual dose. All IMRT plans were verified with MapCHECK (SunNuclear) to ensure 90% pass for detector points with γ index of 3% and 3mm before they were delivered by a 4MV linear accelerator [13,14]. Anteroposterior and lateral x-ray simulation was performed and compared with electronic portal imaging at anteroposterior and lateral directions performed to verify treatment position before the first 3 fractions, 5^th^ fraction and last fraction of IMRT. All patients had anteroposterior and lateral deviations of ≤1.5mm during the whole course of IMRT, justifying the usual practice of giving 2mm margin around GTV to generate PTV in our institution. A separate LOF plan with the isocentres fixed at both lateral bony canthi (blocking the anterior halves of the beams) directed laterally to each side of the orbit and a typical field size of 4.5cm (anteroposterior dimension) by 5.0cm (craniocaudal dimension) was generated. Another 3DCRT plan delivered by 6 beams (one beam directed at gantry angle 270° with a 45-degree wedge, 1 pair of beams at 344° with a 20-degree and a 30-degree dynamic wedges, one pair of beams at 18° with a 20-degree and a 30-degree dynamic wedges and a beam directed at 90°) based on the same CT images were also generated. Both LOF and 3DCRT were produced for each patient for statistical comparison of dosimetric parameters (Figure 1). For 3DCRT and IMRT, though the total radiation dose would not exceed the tolerance dose of the lenses, optic nerves and optic chiasm, weightings were given during optimization procedures in an attempt to eliminate overdose (>107% of prescribed dose) to these structures. Conformity index (CI), homogeneity index (HI) together with dosimetric parameters including the minimum, mean, maximum and median dose, D05 (dose received by the maximal 5% of the target), D01 (dose received by the maximal 1% of the target) of GTV, PTV, globes, lenses, optic nerves, optic chiasm and lacrimal glands were recorded. CI, originally proposed by ICRU 62 and later modified by Paddick, represents the ratio to evaluate the tightness of fit of the Planning Target Volume (PTV) to the prescription isodose volume in treatment plans [15,16]. We applied the formula CI = V_PTV_ × V_TV_/TV^2^_PV_ (V_PTV_: the volume of PTV; V_TV_: the treatment volume encompassed by the prescribed isodose lines; TV_PV_: the volume of the PTV within the prescribed isodose lines) and HI = D_5%_/D_95%_ (D_5%_ and D_95%_: the dose received by the maximal 5% and 95% of the PTV respectively) [17,18]. Unity is considered the most ideal for both CI and HI. The study was carried out in strict compliance with the Helsinki Declaration. Prior approval from local institutional review board was obtained before the study was conducted. Written informed consent was obtained from all patients for publication of this report and any accompanying images.

### Statistical analysis

CI, HI and other dosimetric parameters generated by IMRT as mentioned above were compared with the other two techniques by Mann–Whitney U tests. A two-tailed p-value less than 0.05 was considered statistically significant. All statistical analyses were performed by Statistical Packages for Social Sciences (SPSS) version 19.

## Results

The details of all 10 patients were shown in Table 1. They all completed IMRT for their Graves’ ophthalmopathy without interruption or any acute adverse events. Mean GTV of all patients was 40.92cm^3^ (range: 28.52-50.01cm^3^) while mean PTV was 74.29cm^3^ (range: 54.45-109.22cm^3^).

**Table 1:** Patient characteristics

**Patient**	**Age**	**Sex**	**Date of diagnosis of Graves’ disease**	**Date of diagnosis of Graves’ ophthalmopathy**	**Prior use of systemic steroid**	**Prior corrective eye operation**	**Prior radio- iodine therapy**	**Time between diagnosis to radiotherapy (months)**	**Use of systemic steroid during radiotherapy**	**Use of anti-thyroid drugs during radiotherapy**
1	24	F	07/2007	10/2008	Y	N	N	14	Y	CMZ
2	54	F	09/2001	11/2008	Y	N	Twice	8	Y	N
3	36	F	03/2010	08/2010	Y	N	N	3	N	N
4	43	M	01/1995	01/1995	Y	N	N	180	Y	N
5	31	M	06/2009	06/2009	Y	N	N	8	Y	N
6	46	M	07/2009	07/2009	Y	Y	N	12	Y	N
7	72	M	07/2008	07/2008	Y	N	N	10	Y	PTU
8	47	F	01/1996	03/1996	Y	Y	N	156	Y	N
9	39	M	05/2009	05/2009	Y	N	N	8	Y	N
10	46	F	01/2003	07/2009	Y	N	N	4	N	PTU

CMZ – carbimazole, PTU- propylthiouracil.

### CI and HI

Mean CI generated by IMRT was 1.24 (range: 1.15-1.30) while those for 3DCRT and LOF were 1.74 (range: 1.42-1.90) and 3.11 (range: 2.42-4.27) respectively. When IMRT was compared with 3DCRT, a superior CI was observed in IMRT (p=0.005). Likewise when IMRT was compared with LOF, IMRT definitely resulted in a better CI (p=0.005). Mean HI generated by IMRT, 3DCRT and LOF were 1.05 (1.03-1.08), 1.08 (1.05-1.14), 1.60 (1.06-4.60) respectively, with significant statistical difference in favor of IMRT when compared with 3DCRT (p=0.007) and LOF (p=0.005).

### Comparison of dosimetric parameters of targets (Table 2)

#### GTV

##### IMRT versus 3DCRT

When GTV of left and right eye were considered individually, both the left and right eyes received a significantly higher minimum (p=0.005 for both left and right eyes) with IMRT. When they were combined together to form a single GTV, again IMRT offered less cold spots than 3DCRT as reflected by a higher minimum dose received by the GTV (p=0.005) (Table 2).

**Table 2:** Dosimetric parameters of GTV and PTV planned by three different treatment techniques

**Parameters (mean[range], Gy)**	**LOF**	**3DCRT**	**IMRT**
*GTV*			
Minimum	5.03 [0.46-9.28]	7.30 [5.43-9.54]	9.85 [9.56-9.98]**,##
Maximum	10.66 [10.58-10.86]	10.61 [10.55-10.68]	10.72 [10.52-11.03]
Mean	10.09 [9.49-10.27]	10.30 [10.27-10.34]	10.32 [10.27-10.44]##
Median	10.27 [10.11-10.36]	10.34 [10.30-10.37]	10.31 [10.27-10.44]
D05	10.50 [10.37-10.59]	10.50 [10.46-10.54]	10.49 [10.41-10.69]
D01	10.57 [10.47-10.73]	10.54 [10.50-10.60]	10.56 [10.45-10.80]
*PTV*			
Minimum	3.77 [0.35-7.95]	5.72 [3.98-8.53]	8.61 [6.85-9.53]**,##
Maximum	10.67 [10.59-10.87]	10.61 [10.56-10.69]	10.76 [10.52-11.03]*
Mean	9.86 [8.85-10.21]	10.19 [10.10-10.24]	10.26 [10.20-10.35]**,##
Median	10.23 [10.07-10.58]	10.28 [10.20-10.32]	10.28 [10.23-10.39]
D05	10.49 [10.39-10.59]	10.49 [10.45-10.53]	10.47 [10.40-10.67]
D01	10.57 [10.49-10.72]	10.54 [10.50-10.61]	10.55 [10.44-10.78]

* p<0.05, ** p<0.01 compared with 3DCRT.

# p<0.05, ## p<0.01 compared with LOF.

##### IMRT versus LOF

The GTV of both eyes, when considered individually, received a higher minimum (left: p=0.005; right p=0.005) and a mean dose (left: p=0.008; right: p=0.005) with IMRT. When added together to form single GTV of both eyes, IMRT was able to give a higher minimum and mean dose (p=0.005 for both respectively).

#### PTV

##### IMRT versus 3DCRT

When dividing PTV according to individual eye, both the left and right eyes had a higher minimum (p=0.005 for both eyes) and mean dose (left: p=0.007; right: p=0.005) with IMRT. When they were combined together to be a single PTV, a higher minimum (p<0.001), maximum (p=0.022) and mean dose (p=0.007) could be achieved by IMRT.

##### IMRT versus LOF

When PTV was considered individually, both the left and right eyes received a higher minimum (p=0.005 for both eyes) and mean dose (left: p=0.007; right: p=0.005) with IMRT. Apart from that, the right eyes also received a higher median dose (p=0.005). When they were combined together to become a single PTV, a higher minimum and mean dose (p=0.005 for both) could be observed when planned with IMRT.

### Comparison of dosimetric parameters of OARs (Table 3)

#### Globes

##### IMRT vs 3DCRT

IMRT provided a better sparing to the globes compared with 3DCRT. The left globes received a reduced mean (p=0.007), median (p=0.008) and D05 (p=0.041) with IMRT. Similarly, the right globes were also better spared with unnecessary irradiation with IMRT, with a reduced minimum (p=0.009), mean (p=0.009), median (p=0.012) and D05 (p=0.028) (Table 3).

**Table 3:** Dosimetric parameters of OARs planned by three radiation treatment techniques

**Parameters (mean[range], Gy)**	**LOF**	**3DCRT**	**IMRT**
*Left globe*			
Minimum	1.16 [0–6.64]	2.88 [0.59-4.35]	3.17 [1.23-9.98]
Maximum	10.56 [10.47-10.74]	10.57 [10.48-10.69]	10.57 [10.34-11.03]
Mean	7.53 [5.60-10.10]	9.08 [8.26-9.53]	8.55 [7.54-10.32]**
Median	8.33 [5.80-10.23]	9.96 [9.19-10.64]	9.26 [8.15-10.30]**
D05	10.39 [10.15-10.53]	10.47 [10.29-10.61]	10.34 [10.20-10.53]*
D01	9.48 [0.44-10.66]	10.00 [5.30-10.65]	10.44 [10.27-10.73]
*Right globe*			
Minimum	1.48 [0.21-7.31]	3.35 [1.95-4.57]	2.27 [0.55-3.93]**
Maximum	10.55 [10.45-10.74]	10.55[10.46-10.64]	10.51 [10.37-10.89]
Mean	7.66 [5.98-10.06]	9.09 [8.41-9.74]	8.37 [7.27-9.09]**
Median	8.51 [6.35-10.16]	9.86 [9.29-10.29]	9.23 [7.84-9.93]*
D05	10.37 [10.19-10.51]	10.44 [10.27-10.55]	10.31 [10.22-10.55]*
D01	10.47 [10.31-10.65]	10.50 [10.37-10.59]	10.40 [10.27-10.71]
*Left lens*			
Minimum	1.91 [0.43-8.23]	4.51 [3.82-4.88]	3.06 [1.86-4.39]**
Maximum	4.93 [1.92-10.04]	7.47 [7.13-8.20]	5.67 [3.82-7.64]**
Mean	3.12 [0.88-9.65]	5.71 [5.33-6.25]	4.14 [2.62-5.60]**
Median	3.05 [0.85-9.84]	5.69 [5.18-6.20]	4.09 [2.46-5.55]**
D05	4.26 [1.47-10.03]	6.87 [6.58-7.33]	5.15 [3.53-7.15]*
D01	4.59 [1.91-10.04]	7.20 [6.89-7.77]	5.43 [3.64-7.44]**
*Right lens*			
Minimum	2.01 [0.39-8.22]	4.52 [3.70-5.14]	3.05 [1.86-4.32]**
Maximum	4.94 [2.18-9.91]	7.41 [6.68-7.93]	5.59 [3.79-7.78]*
Mean	3.24 [1.05-9.49]	5.77 [5.20-6.42]	4.12 [2.57-5.46]**
Median	3.13 [0.91-9.49]	5.74 [5.15-6.45]	4.07 [2.48-5.45]**
D05	4.45 [2.15-9.83]	6.90 [6.26-7.38]	4.93 [3.37-7.03]*
D01	4.76 [2.17-9.89]	7.21 [6.54-7.71]	5.39 [3.57-7.53]*
*Left optic nerve*			
Minimum	9.68 [9.24-10.24]	9.88 [9.17-10.24]	9.99 [9.76-10.16]#
Maximum	10.51 [10.41-10.59]	10.51 [10.46-10.55]	10.42 [10.22-10.57]#
Mean	10.29 [10.09-10.43]	10.33 [10.23-10.39]	10.25 [10.10-10.36]
Median	10.32 [10.11-10.43]	10.34 [10.27-10.40]	10.25 [10.11-10.38]*
D05	10.46 [10.30-10.55]	10.45 [10.38-10.51]	10.35 [10.17-10.49]*,#
D01	10.49 [10.36-10.51]	10.48 [10.41-10.52]	10.38 [10.17-10.53]#
*Right optic nerve*			
Minimum	9.82 [9.25-10.31]	10.04 [9.83-10.28]	10.08 [9.93-10.22]
Maximum	10.51 [10.35-10.62]	10.51 [10.43-10.58]	10.49 [10.27-10.76]
Mean	10.31 [10.14-10.46]	10.35 [10.24-10.40]	10.26 [10.08-10.36]*
Median	10.34 [10.20-10.45]	10.37 [10.27-10.46]	10.26 [10.08-10.36]*,#
D05	10.46 [10.30-10.57]	10.47 [10.38-10.54]	10.39 [10.18-10.53]#
D01	10.49 [10.32-10.60]	10.49 [10.41-10.57]	10.44 [10.22-10.67]
*Optic chiasm*			
Minimum	0.28 [0.09-0.68]	2.30 [1.84-3.01]	3.38 [0.99-9.76]##
Maximum	6.72 [0.41-9.84]	8.30 [5.13-9.65]	8.32 [3.60-10.35]
Mean	1.57 [0.16-3.49]	3.94 [2.67-5.64]	4.84 [1.44-10.15]##
Median	1.02 [0.14-3.11]	3.46 [2.52-5.55]	4.55 [1.35-10.14]##
D05	4.71 [0.29-7.97]	6.76 [3.78-8.29]	6.95 [2.19-10.24]#
D01	6.01 [0.35-9.39]	7.72 [4.56-9.10]	7.68 [2.84-10.29]
*Left lacrimal gland*			
Minimum	3.63 [0.79-8.98]	5.88 [3.62-7.37]	6.24 [4.18-8.39]#
Maximum	9.27 [0.86-10.56]	10.43 [9.96-10.68]	10.52 [10.20-10.84]#
Mean	8.06 [5.84-10.03]	9.11 [7.73-9.80]	9.25 [7.94-10.28]#
Median	8.32 [5.78-10.06]	9.34 [8.00-9.96]	9.46 [8.04-10.34]#
D05	9.97 [9.27-10.75]	10.23 [9.65-10.60]	10.34 [9.81-10.66]#
D01	10.13 [9.48-10.82]	10.36 [9.83-10.65]	10.44 [10.00-10.81]#
*Right lacrimal gland*			
Minimum	3.57 [0.85-8.99]	5.36 [3.07-7.12]	5.21 [3.11-7.33]
Maximum	10.28 [9.83-10.81]	10.35 [9.77-10.61]	10.44 [10.14-10.74]
Mean	8.18 [5.67-9.92]	8.89 [7.64-9.87]	8.94 [6.87-10.17]
Median	8.54 [5.84-9.95]	9.14 [7.87-10.22]	10.26 [6.81-10.30]
D05	9.99 [9.08-10.74]	10.15 [9.41-10.52]	10.29 [9.89-10.58]#
D01	10.16 [9.50-10.78]	10.28 [9.59-10.56]	10.39 [10.09-10.63]#

* p<0.05, ** p<0.01 compared with 3DCRT.

# p<0.05, ## p<0.01 compared with LOF.

##### IMRT vs LOF

There was no statistical difference in all dosimetric parameters for both globes when comparing IMRT versus LOF. It was originally thought that LOF, as compared with IMRT, would contribute less radiation to the globes as their anterior portions were not within the radiation portals of LOF, it was not demonstrated in our study however. The reason was that the anterior thirds of the globes still received some dose owing to radiation falloff and scattered radiation in LOF. This gave rise to the result that, despite lower value of parameters generated by LOF, they were not statistically different from those generated by IMRT.

#### Lenses

##### IMRT versus 3DCRT

Similar to the globes, IMRT definitely produced better sparing to the both lenses compared with 3DCRT, with a reduced minimum (p=0.005 both lenses), maximum (left: p=0.009; right: p=0.013), mean (left: p=0.005; right: p=0.007), median (left: p=0.005; right: p=0.007), D05 (p=0.013 for both lenses) and D01 (left: p=0.009; right: p=0.017).

##### IMRT versus LOF

There was no difference in all dosimetric parameters for both lenses between IMRT and LOF. Similar to the globes, though the lenses were not included in the radiation portals of LOF, they still received certain radiation due to falloff and scattered radiation. This resulted in numerically lower values of the parameters achieved by LOF, but statistical differences were not found when compared with IMRT.

#### Optic nerves

##### IMRT versus 3DCRT

IMRT produced some dose sparing to both the left and right optic nerves. The median dose (p=0.028) and D05 (p=0.038) of left optic nerve was slightly reduced and the mean (p=0.037) and median dose (p=0.021) of the right optic nerve was also slightly improved when planned by IMRT.

##### IMRT versus LOF

IMRT produced a slightly higher minimum dose to the optic nerves (left: p=0.022; right: p=0.110) when compared with LOF, simply because the optic nerves were totally encompassed by the PTV. The improved coverage of PTV inevitably led to an increased minimum dose to the optic nerves. However weightings were given to the optic nerves during IMRT optimization so that hot spots did not fall within them. It was reflected by a lower maximum dose (p=0.038), D05 (p=0.011) and D01 (p=0.028) to the left optic nerve and a lower median dose (p=0.037) and D05 (p=0.047) to the right optic nerve.

#### Optic chiasm

##### IMRT versus 3DCRT

There were no significant statistical differences in the dosimetric parameters of the optic chiasm between IMRT and 3DCRT, though they were all slightly higher when planned by IMRT.

##### IMRT versus LOF

IMRT produced a higher minimum (p=0.005), mean (p=0.007), median (p=0.007), D05 (p=0.022) to the optic chiasm as compared with LOF. This was because the IMRT beams directed from posterior to anterior contributed to the increased dose to the optic chiasm in contrast to the steep drop of radiation dose in that region delivered by LOF.

#### Lacrimal glands

##### IMRT versus 3DCRT

There were no significant statistical differences in the dosimetric parameters of the both lacrimal glands between IMRT and 3DCRT, though again they were all slightly higher when planned by IMRT.

##### IMRT versus LOF

IMRT produced a higher minimum (p=0.017), maximum (p=0.013), mean (p=0.022) and median dose (p=0.047) as well as D05 (p=0.047) and D01 (p=0.047) to the left lacrimal glands. Similarly, D05 (p=0.047) and D01 (p=0.041) of the right lacrimal glands were also higher with IMRT. The lacrimal glands, as situated anteriorly in the globe at the upper outer quadrants of the conjunctiva, were not within the radiation portals when planned by LOF. As the most anterior portions of the globes were blocked from radiation in LOF, the lacrimal glands, as a result, were also blocked from radiation as well. The dose to the lacrimal glands was thus lower with LOF.

### Planning time, treatment time and monitor units

The average planning time for an IMRT plan by our experienced physicists and dosimetrists was around 2 hours, as compared with 1.5 hours and 30 minutes for a 3DCRT and LOF plan respectively. Similarly treatment time of each fraction for IMRT was also longer (26 minutes) than that delivered for a 3DCRT (16 minutes) and LOF (10 minutes). Average monitor units (MU) consumed was greater for IMRT (773 MU), in contrast to that for 3DCRT (252 MU) and LOF (197 MU).

### Radiation-induced cataract based on NTCP model

The lenses are the only potential critical organs which might suffer from radiotherapy-induced complications like cataract, since we only delivered a low dose of radiation to the orbits. We calculated their normal tissue complication probability (NTCP) after treatment with each radiation technique, based on the equivalent uniform dose (EUD) and the following formula: $NTCP=\frac{1}{1+{\left(\frac{TD_{50}}{EUD}\right)}^{4\gamma_{50}}}$, where *TD*_*50*_ is the tolerance dose for a 50% complication rate at a specific time interval when the whole organ of interest is homogeneously irradiated and γ_50_ is a model parameter that is specific to the normal tissue and describes the slope of the dose response curve [19-21]. Ranges of values NTCP of the left lens contributed by LOF, 3DCRT and IMRT were 0.003-3.194, 0.213-0.429 and 0.010-0.280 respectively. Similarly NTCP for the right lens contributed by LOF, 3DCRT and IMRT were 0.001-2.942, 0.188-0.480 and 0.009-0.236 respectively. Statistical comparison demonstrated that NTCP contributed by IMRT was higher than that contributed 3DCRT (left: p=0.001; right: p=0.013) but not higher than that by LOF (left: p=0.096; right: p=0.257), revealing the risk of radiation-induced cataract might be higher for IMRT as compared 3DCRT but not with LOF.

## Discussion

Graves’ ophthalmopathy is the commonest extrathryoidal manifestation of Graves’ disease [1,2,22]. Clinical presentations include proptosis, eyelid swelling and retraction, chemosis, compressive optic neuropathy and papilloedema. The underlying pathogenesis is believed to be autoimmune-related leading to excessive infiltration of lymphocytes and excessive production of hydrophilic glycosaminoglycans and subsequently expansion of retro-orbital tissues and enlargement of extraocular muscles. Treatment options include steroid therapy, corrective/decompressive surgery, radiation therapy or combination of these approaches [3-5,23]. Orbital irradiation has been practiced for more than 60 years. It is usually reserved for cases with moderate and severe degree of exophthalmos or compressive optic neuropathy when decompressive surgery is not immediately available or if patients are medically contraindicated for surgery. Though producing conflicting and controversial results as demonstrated in several double-blind randomized-controlled trials and numerous non-randomized trials with a small potential risk of long-term adverse events, orbital irradiation is still an acceptable treatment option for such disease [24-29]. There are also disputes on the optimal dose of orbital irradiation [30,31]. For studies in favor of orbital irradiation, they used delivered 20Gy in 10 fractions over 2 weeks as the standard dose while more recent studies demonstrated that a lower dose was also equally effective. A randomized study revealed treatment with 20Gy of 1Gy-fraction weekly over 20 weeks was more effective and better tolerated than treatment with 20Gy of 2Gy-fraction over 2 weeks and 10Gy of 1Gy-fraction over 2 weeks [30]. The dose prescription in our study was based on this study after taking the balance between the total dose and the total treatment duration into consideration. LOF technique has been employed by radiation oncologists for many decades in virtue of its simple and swift set-up procedures. However the obvious drawbacks of this technique are insufficient dose to the insertions of the extraocular muscles and the most anterior portion of the retro-orbital fat which are commonly involved in Graves’ ophthalmopathy, as well as inhomogeneous dose distribution within the target, since the anterior portion of the globes are usually blocked from the radiation portals of LOF in order to reduce the dose to the lenses. 3DCRT and IMRT, widely adopted for more than 10 years, were regarded as the current standard radiation treatment technique for head and neck and orbital tumors [6-10]. However there has been so far no study regarding the use of IMRT as the treatment technique for Graves’ ophthalmopathy and only a few studies of using IMRT for other orbital diseases [6,11,12].

**Figure 2:**
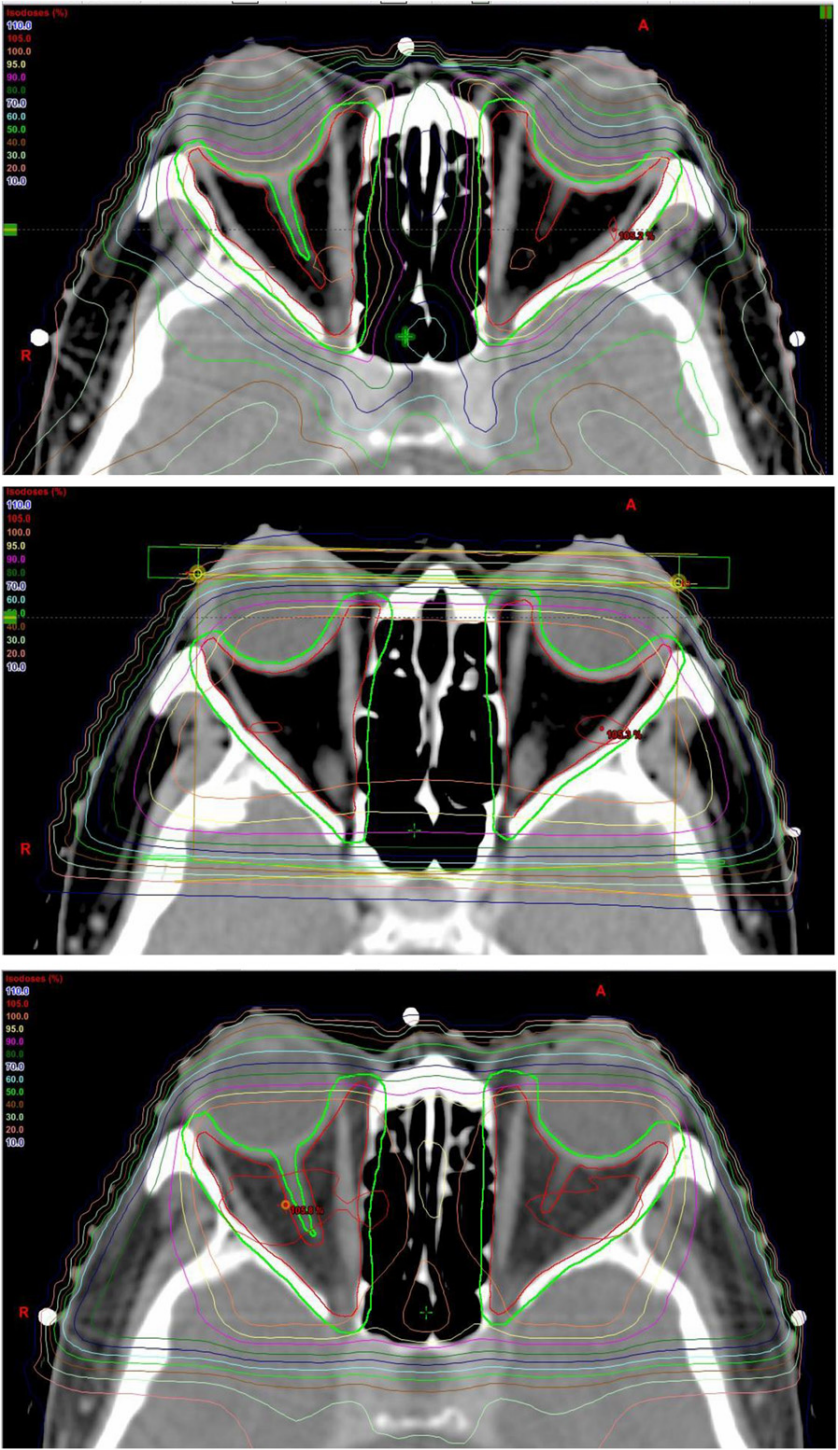
Dose distribution and target coverage by IMRT (uppermost), LOF (middle) and 3DCRT (lowermost).

**Figure 3:**
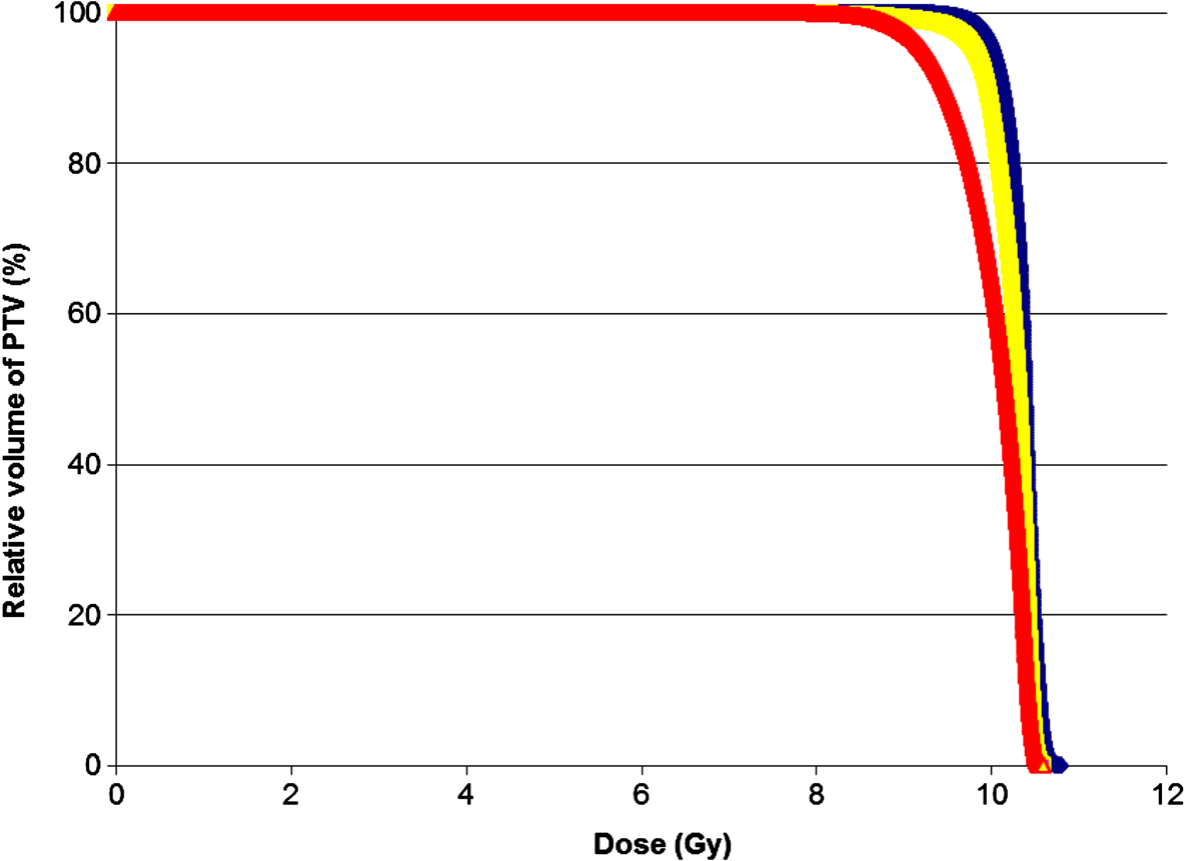
Dose volume histogram of PTV generated by IMRT (blue), 3DCRT (yellow) and LOF (red) in a patient.

In our dosimetric study, we were able to demonstrate that IMRT offered a superior and more conformal coverage of the GTV and PTV compared with 3DCRT and LOF resulting in a better CI (Figure 2). Part of the PTV of our patients was just covered by 70% isodose curves in the plans generated by 3DCRT and LOF while almost 100% of GTV and most of PTV were adequately covered by 95% of prescribed dose in the plans generated by IMRT except at areas close to the ethmoid sinuses where attenuation in the air is low. The superiority of PTV coverage by IMRT could also be reflected by a more favorable dose volume histogram (DVH) in contrast to 3DCRT and LOF (Figure 3). On top of that, more effective elimination of hot and cold spots achieved by IMRT resulted in a more favorable HI. When compared with 3DCRT, IMRT could also better preserve adjacent normal structures including the globes, lenses and the optic nerves in virtue of its better dose fall off and steeper gradient. When comparing IMRT against LOF, IMRT inevitably resulted in a higher dose to the optic nerves and optic chiasm. The resultant increased dose to these structures was due to the extra dose contributed by the posteriorly-oriented beams in IMRT. LOF though could achieve dose sparing to the lacrimal glands as well as to a lesser extent and non-significant sparing to the lenses in our study since they were blocked from its radiation portals, it also compromised the PTV coverage of the insertions of the extraocular muscles and anterior part of the retro-orbital fat. Another drawback of IMRT was the increased risk of radiation-induced complication like cataract as demonstrated in our NTCP model, but the risk was very small and clinically negligible. Having all these considered together, the dosimetric advantages achieved by IMRT still made it an acceptable and encouraging radiation technique despite the longer treatment planning and delivery time, extra monitor units consumed as well as the slightly increased but clinically negligible dose to some nearby normal structures. The clinical outcomes of patients with Graves’ ophthalmopathy may improve after treatment with IMRT owing to its better target coverage especially over the insertions of extraocular muscles and the anterior part of the retro-orbital fat. A long follow up is definitely warranted to observe the clinical outcomes and complications after IMRT.

## Conclusion

IMRT is an acceptable radiation treatment technique for Graves’ ophthalmopathy in virtue of its superior target coverage, better CI and HI, despite an increase in treatment planning and delivery time, consumption of monitor units and a slightly increased but clinically negligible dose to some surrounding structures. It might supersede the other two older radiation techniques when orbital irradiation is contemplated.

## Competing interests

The authors declare that actual or potential conflicts of interest do not exist.

## Authors’ contribution

VHFL, SCYN, CWC, MYL, TWL, GKHA, and DLWK participated in the study design and coordination, performed acquisition of data, and drafted the manuscript. VHFL and CWC participated in the statistical data analysis. All authors reviewed and approved the final manuscript.
